# Two genes encoded by mulberry crinkle leaf virus (MCLV): The *V4* gene enhances viral replication, and the *V5* gene is needed for MCLV infection in *Nicotiana benthamiana*

**DOI:** 10.1016/j.virusres.2023.199288

**Published:** 2023-12-06

**Authors:** Tao-Tao Han, Jia-Xuan Tang, Miao Fang, Peng Zhang, Pei-Yu Han, Zhen-Ni Yin, Yu Ma, Jian Zhang, Quan-You Lu

**Affiliations:** a College of Biotechnology, Jiangsu Key Laboratory of Sericultural Biology and Biotechnology, Jiangsu University of Science and Technology, Zhenjiang, Jiangsu 212100, China; b Key Laboratory of Genetic Improvement of Silkworm and Mulberry, Ministry of Agriculture and Rural Affairs, Sericultural Research Institute, Chinese Academy of Agricultural Sciences, Zhenjiang, Jiangsu 212100, China

**Keywords:** Mulberry crinkle leaf virus, V4, V5, Replication, Host range

## Abstract

Mulberry crinkle leaf virus (MCLV) is a member of the genus *Mulcrilevirus*, family *Geminiviridae*. The expression and functions of the *V4* and *V5* genes encoded by the MCLV genome remain unknown. Here, we confirmed the expression of V4 and V5 by analyzing the V4 and V5 mRNAs and the promoter activity of individual ORFs upstream sequences. The functions of V4 and V5 were investigated by constructing *Agrobacterium*-mediated infectious clones of wild-type MCLV variant П (MCLV vII), MCLV^wt^ and MCLV vП mutants, such as MCLV^mV4^ (start codon of V4 ORF mutated), MCLV^dV4^ (5′-end partial deletion of V4 ORF sequence) and MCLV^mV5^ (V5 ORF start codon mutated). Although MCLV^wt^, MCLV^mV4^, and MCLV^dV4^ could infect natural host mulberry and experimental tomato plants systematically, the replication of the MCLV^mV4^ and MCLV^dV4^ genomes was obviously reduced compared to MCLV^wt^ in both mulberry and tomato plants. MCLV vП expressing V5 could infect *Nicotiana benthamiana* plants systematically, but MCLV^mV5^ could not, implying that V5 is needed for MCLV vП to infect *N. benthamiana* plants. Taken together, V4 is involved in replication of the MCLV genome in host plants, and V5 potentially might extend the host range. Our findings lay a foundation for in-depth insight into the functions of MCLV-encoded proteins and provide a novel perspective for the subsequent study of MCLV-host plant interactions.

## Highlights

• *V4* and *V5* genes encoded by MCLV vII could be transcribed and expressed normally.
• *V4* gene is involved in replication of MCLV genome in host plants.
• *V5* gene is required for MCLV infection in *Nicotiana benthamiana, V5* gene has potential function of extending host range.

## 1 Introduction

Geminiviruses are a group of nonenveloped plant viruses with a genome consisting of one or two circular single-stranded DNAs (ssDNAs) with a size of 2.5–5.2 kb (Fiallo-Olivé et al., 2021). Geminiviruses infect economically important crops worldwide and cause severe losses in agriculture (Devendran et al., 2022; Rojas et al., 2018). The family *Geminiviridae* comprises more than 520 plant virus species. They are assigned to 14 genera based on their insect vectors, genome organization, and host range. These genera include *Becurtovirus, Begomovirus, Capulavirus, Citlodavirus, Curtovirus, Eragrovirus, Grablovirus, Maldovirus, Mastrevirus, Mulcrilevirus, Opunvirus, Topilevirus, Topocuvirus,* and *Turncurtovirus* (Fiallo-Olivé et al., 2021; Roumagnac et al., 2021). As with many viruses (Callaway et al., 2001; Faust et al., 2017; Weber and Bujarski 2015), geminiviruses encode few genes due to their small genomes (Hanley-Bowdoin et al., 2013), and the proteins encoded by these genes often play multiple roles during viral infection (Luna and Lozano-Duran 2020). For example, the V3 protein of mulberry crinkle leaf virus (MCLV) functions not only as a putative movement protein (Lu et al., 2015) but also as a pathogenicity determinant and posttranscript gene silencing suppressor (Lu et al., 2019; Lu et al., 2020).

Viral proteins are essential factors for viruses to complete their life cycles in hosts and disseminate by insect vectors. Understanding the functions of viral proteins not only contributes to virus biology, such as viral pathogenicity, genome replication, or host ranges but also promotes the development of antiviral breeding. However, there are limited studies on the functions of viral proteins encoded by geminivirus species (especially some newly discovered geminivirus species), for instance, the V2 and V3 proteins of passion fruit chlorotic mottle virus (PCMoV), a member of the genus *Citlodavirus* (Fontenele et al., 2018), whose functions are completely unclear. More functions of some geminivirus-encoded proteins are constantly being discovered. For example, the C4 protein of tomato leaf curl virus (ToLCV), a member of the genus *Begomoviurs*, is considered a pathogenicity determinant (Mei et al., 2018a; Rigden et al., 1994) and gene silencing suppressor (Mei et al., 2018b). In addition, C4 also plays roles in suppressing hypersensitive response (HR) and salicylic acid (SA)-mediated defense as well as conferring drought stress tolerance independent of abscisic acid in plants (Corrales-Gutierrez et al., 2020; Medina-Puche et al., 2020; Mei et al., 2020). Recently, several geminivirus proteins with molecular weights below 10 kDa have been discovered to perform roles in biological functions. For example, V3 (∼8.6 kDa) and C5 (∼7.58 kDa) of tomato yellow leafroll virus (TYLCV), in which V3 not only acts as an RNA silencing suppressor but also plays a role in promoting viral intercellular movement, and C5 serve as a pathogenic determinant (Gong et al., 2021; Gong et al., 2022; Zhao et al., 2022).

**Fig. 1:**
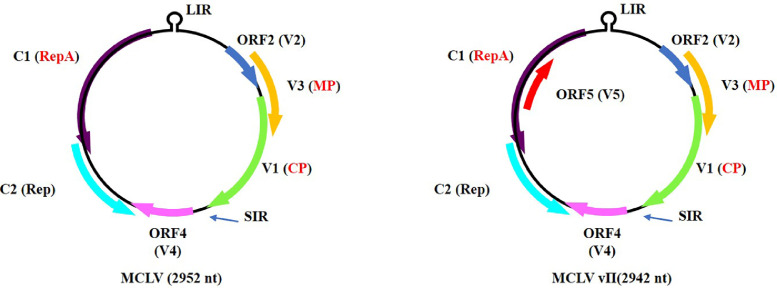
Schematic representation of the genome organization of two MCLVs.

MCLV, a geminivirus identified in mulberry trees (Lu et al., 2015; Ma et al., 2015) belonging to the genus *Mulcrileviru*s, family *Geminiviridae* (Roumagnac et al., 2021), naturally infects mulberry trees only thus far (Han et al., 2022; Lu et al., 2022) and is transmitted by a species of leafhopper known as *Tautoneura mori* (Lu et al., 2022). The MCLV genome contains 6 open reading frames (ORFs) (Figs. 1 left). In the viral sense, the V1, V2, V3 and V4 proteins are encoded by four ORFs. V1 was identified as a viral coat protein (CP), and V3 was determined to be a viral movement protein (MP), and also functions as a posttranscriptional gene silencing suppressor and a pathogenicity determinant (Lu et al., 2019; Lu et al., 2020; Yang et al., 2018). However, the roles of the V2 and V4 proteins remain to be clarified. In addition, the C1 and C2 proteins of MCLV are involved in replication and are encoded by two ORFs in the complementary sense (Lu et al., 2015). Interestingly, another MCLV variant (temporarily named MCLV vII in this study) was discovered by genetic diversity analysis of MCLV in naturally infected mulberry trees (Yu 2021). One of the most distinguishing differences between MCLV vII (Figs. 1 right) and MCLV (Figs. 1 left) is that MCLV vII has one more ORF in the viral sense, referred to as ORF5. ORF5 contains 309 nucleotides (nt) and is fully incorporated into the C1 ORF, which has 796 nt (Figs. 1 right). However, whether ORF5 is transcribed into mRNA and encodes a functional protein during MCLV infection remains to be elucidated.

The prerequisite for a viral protein to have biological functions is that it can be expressed in the context of viral infection. In this study, we used reverse transcription PCR (RT‒PCR) and promoter activity assays to determine the transcription and expression of the MCLV vII *V4* and *V5* genes. By constructing MCLV vII-based mutants MCLV^mV4^, MCLV^dV4^ and MCLV^mV5^, we discovered that V4 is crucial for MCLV genome replication and that the deletion of V4 significantly reduces the accumulation of the MCLV genome. Additionally, V5 is needed for MCLV vII infection of *N. benthamiana*. In the absence of V5, MCLV vII cannot infect *N. benthamiana*. This study established the groundwork for future MCLV research and application by revealing new perspectives about the roles of MCLV gene products.

## 2 Materials and methods

### 2.1 Preparation of plant materials

To obtain virus-free mulberry plants, we collected seeds from mulberry (Yu711, a cultivar susceptible to bacterial and viral diseases) that had neither virus infection-like symptoms nor virus infection determined by high-throughput sequencing. Healthy mulberry seedlings were obtained from the collected seeds upon cultivation in a temperature- and light-controlled room (26 °C, 16/8 h light/dark photoperiod). The seedlings with 3 fully expanding leaves were transplanted into 12 cm diameter pots filled with the soil substrate, with one seedling per pot.

*Nicotiana benthamiana* and tomato plants were grown in a growth chamber at 25 °C constant temperature, 50–60% relative humidity, and a 16/8 h light/dark photoperiod.

### 2.2 Virus source and mutant construction

The MCLV vII identified from naturally infected mulberry was used as wild-type (WT) virus in this study. The full-length sequence of the MCLV vII genome DNA has been determined previously and submitted to the GenBank database with the accession number MZ420357 (Yu 2021). An *Agrobacterium*-mediated infectious clone of the MCLV vII genome (pCA-1.1MCLV^WT^) was previously constructed by our laboratory. The construction contains a duplicated large intergenic region (LIR) and one copy of the sequence flanking the LIR of the MCLV^WT^ genome. In the systemic leaves of mulberry plants agroinoculated with pCA-1.1MCLV^WT^, genome-sized MCLV vII DNA molecules were detected, confirming that the construction is available (Han et al., 2022).

The expression vector pCAMBIA1391z, which contains a β-glucuronidase (GUS) reporter gene lacking a promoter, was used in this study. To generate the constructs used for analyzing V4 and V5 promoter activity, we designed two sets of primers, pCA V4p F1/pCA V4p R and pCA V4p F2/pCA V4p R (Tables S1), for PCR amplification of 776 nt and 576 nt sequences upstream of the V4 ORF and three sets of primers, pCA V5p F1/pCA V5p R, pCA V5p F2/pCA V5p R, and pCA V5p F3/pCA V5p (Tables S1), for PCR amplification of 938 nt, 838 nt and 738 nt sequences upstream of the V5 ORF, respectively. The target fragments were introduced into the *Sal* І-digested pCAMBIA1391z vector by a seamless cloning kit, ClonExpress MultiS One Step Cloning Kit (Vazyme, Nanjing, China), as specified by the supplier. These five constructs were transformed into *Escherichia coli* strain Top10, verified by sequencing (Shangya Biotechnology Co., Ltd., Hangzhou, China) and named pV4–1-GUS, pV4–2-GUS, pV5–1-GUS, pV5–2-GUS, and pV5–3-GUS, respectively. *Agrobacterium*-mediated pCAMBIA1391z-CaMV 35S (p35S-GUS) constructed previously (Smith et al., 2022) was used as a positive control.

To study the function of MCLV vII V4 and V5, the infectious clones of mutant viruses MCLV^mV4^, MCLV^dV4^, and MCLV^mV5^ were constructed using pCA-1.1MCLV^WT^ as the backbone. For MCLV^mV4^, the start codon (AUG) of the V4 ORF was mutated to UUG to create a V4 ORF-deficient mutant virus. Because the last 39 nt (343 nt-381 nt) sequence of the V4 ORF (total 381 nt) overlaps with the C2 ORF and the second 27 nt (316 nt-342 nt) sequence is the C2 ORF transcription termination site predicted by Term-PseKNC (http://lin-group.cn/server/iTerm-PseKNC/predictor.php), to guarantee the normal transcription of C2, we deleted only the first 315 nt in the V4 ORF sequence to create the MCLV vII ORF4 deletion mutant MCLV^dV4^. For MCLV^mV5^, the start codon (AUG) of the V5 ORF was mutated to GTG, which is a synonymous mutation of C1, to avoid interfering with the normal expression of amino acids at the same position of C1.

The nucleotide sequences used to construct *Agrobacterium*-mediated infectious clones of MCLV^mV4^, MCLV^dV4^, and MCLV^mV5^ were amplified from pCA-1.1MCLV^wt^ using primers MCLV-F1/MCLV-mV4 R1 and MCLV-mV4 F2/MCLV-R2; mCLV-F1/MCLV-dV4 R1 and MCLV-dV4 F2/MCLV-R2; and mCLV-F1/MCLV-mV5 R1 and MCLV-mV5 F2/MCLV-R2 (Tables S1), respectively. The target fragments were introduced into *Sal* I-digestion pCAMBIA2300 by seamless cloning kit, ClonExpress MultiS One Step Cloning Kit (Vazyme). These three constructs were transformed into *E.* coli strain Top10, verified by sequencing (Shangya Biotechnology Co., Ltd.) and named pCA-MCLV^mV4^, pCA-MCLV^dV4^, and pCA-MCLV^mV5^.

It has been revealed that mixed inoculation of two severely defective maize streak virus (MSV) chimeric infectious clones can restore the systemic infection of the virus and produce typical symptoms (Monjane et al., 2014). Therefore, based on the above experimental approaches (Monjane et al., 2014) and homologous recombination repair mechanism (Li et al., 2019b), we used the specific primers V5 F/V5 R to amplify the full-length V5 coding sequence (Tables S1) and cloned it into the pRI 101 AN vector (TaKaRa, Beijing, China) to generate a recombinant plasmid, pRI-V5, which expressed V5 under the 35S promoter. This construct was transformed into *E. coli* strain Top10 and verified by sequencing (Shangya Biotechnology Co., Ltd.).

A pair of specific primers, MCLV cjF/MCLV cjR (Tables S1), were synthesized according to the CP gene sequence of MCLV vII for detection of the presence of MCLV in experimental materials.

All primers used in this study were synthesized by GenScript Biotech Co., Ltd. (Nanjing, China).

### 2.3 Preparation of inoculation samples

All recombinant plasmids, including pV4–1-GUS, pV4–2-GUS, pV5–1-GUS, pV5–2-GUS, pV5–3-GUS, pCA-MCLV^mV4^, pCA-MCLV^dV4^, pCA-MCLV^mV5^, and pRI-V5, were extracted from *E. coli* transformed with the respective plasmids and transformed into *Agrobacterium tumefaciens* EHA105 competent cells by electroporation. *Agrobacteria* with the recombinant plasmids were cultured in Luria–Bertani (LB) medium with a final concentration of 50 μg/ml kanamycin and 50 μg/ml rifampicin at 28 °C in a shaker at a speed of 200 rpm. Cultivated *Agrobacterium* cells were collected by centrifugation at 7000 rpm for 2 min and resuspended to a final concentration of OD_600_=1.0 in a buffer containing 0.5% (W/V) d-glucose, 50 mM 2-(N-morpholino)-ethane sulfonic acid (MES, pH 5.6), 2 mM Na_2_PO_4_, and 100 μM acetosyringone. The resuspended *Agrobacterium* cultures were incubated at room temperature for 3 h before inoculation.

### 2.4 Protoplast isolation and transfection

Protoplasts were isolated from 3- to 4-week-old and healthy *N. benthamiana* plants and transfected with virus plasmids pCA-1.1MCLV^WT^, pCA-MCLV^mV4^, and pCA-MCLV^dV4^ as previously described (Dai and Wang 2022) with slight modifications. Briefly, sliced 5 gram leaves (remove the petioles and midribs) sterilized by 0.2% sodium hypochlorite were treated with 0.5 M mannitol for 10 min at 25 °C. After removing the mannitol solution, these leaves were digested with 20 ml of enzyme solution containing 1% Cellulase R10 (Yakult Honsha, Tokyo, Japan), 0.5% Macerozyme R10 (Yakult), 0.45 Mannitol, and 20 mM MES (pH 5.7) for 5 h at 25 °C. Protoplasts were collected by centrifugation for 5 min at 650 rpm and resuspended in washing solution containing 154 mM NaCl, 125 mM CaCl_2_, 5 mM KCl, 5 mM glucose, and 2 mM MES (pH 5.7). The obtained protoplasts were observed and counted using a hemocytometer plate under a microscope (OLYMPUS BX53F, Japan) (Figures S1). The accumulation levels of viruses in protoplast cells were estimated using growth multiples detected by qPCR described in 2.8. Growth multiples were calculated by the formula, growth multiple = copy number of 24 h −0 h/0 h posttransfection.

### 2.5 Inoculation with the infectious clones

#### 2.5.1 For *N. benthamiana* plants

*Agrobacterium*-mediated infectious clones (pCA-1.1MCLV^WT^, pCA-MCLV^mV5^) were inoculated into *N. benthamiana* plants as previously described (Lu et al., 2022). Simultaneously, *N. benthamiana* plants inoculated with empty pCAmbin2300 vector were used as the negative control.

#### 2.5.2 For tomato seedlings

The infectious clones (*Agrobacterium*-containing pCA-MCLV^mV4^, pCA-MCLV ^dV4^ and pCA-1.1MCLV^WT^) were inoculated into the stems of the *ca.* 12 cm tall tomato plants with a 1 ml syringe as described previously (Han et al., 2022). Simultaneously, tomato plants inoculated with empty pCAmbin2300 vector were used as the negative control.

#### 2.5.3 For young mulberry seedlings

Before agroinfiltration, the prepared healthy mulberry seedlings were tested for MCLV vII by PCR with MCLV vII-specific primers MCLV cjF/MCLV cjR (Tables S1). The inoculation of the infectious clones (*Agrobacterium*-containing pCA-MCLV^mV4^, pCA-MCLV^dV4^ and pCA-1.1MCLV^WT^) into mulberry plants was carried out using the modified “young leaf-stab inoculation” method described previously (Lu et al., 2022). Simultaneously, mulberry plants inoculated with empty pCAmbin2300 vector were used as the negative control.

The inoculated plants were first placed in the dark for 18 h and then grown in a growth chamber at a constant temperature of 25 °C with a 16 h light, 8 h dark photoperiod. The inoculation experiment was repeated 4 times.

### 2.6 GUS fluorometric assays

GUS fluorometric assays were performed as described previously (Smith et al., 2022). Briefly, *Agrobacterium* containing transient expression vectors pV4–1-GUS, pV4–2-GUS, pV5–1-GUS, pV5–2-GUS, pV5–3-GUS, empty pCAmbin1391z vector (negative control), and p35S-GUS (positive control) were infiltrated into *N. benthamiana* leaves. The infiltrated leaf patches were collected 72 h post infiltration (hpi) to test GUS activity (Smith et al., 2022). The promoter activity was estimated by the measured fluorescence value of GUS in the different samples. The assay was repeated 3 times.

### 2.7 DNA and RNA extraction and RT‒PCR

Total RNA and DNA were extracted from plant leaves (∼0.1 g) and protoplast cells using RNAisoPlus (TaKaRa) and EZ-10 Spin Column Plant Genomic DNA Purification Kit (Sangon Biotech, Shanghai, China), respectively, according to the manufacturer's instructions. The extracted DNA was used as a template for PCR amplification using Premix Taq™ (TaKaRa) to confirm the presence or absence of MCLV in the collected samples. The extracted RNAs were digested with DNase I (TaKaRa) and then reverse-transcribed using a Reverse Transcriptase M-MLV (RNase H-) kit (TaKaRa) and a gene-specific reverse primer. The cDNAs were amplified by PCR using specific primers for target genes.

### 2.8 Quantitative real-time PCR (qPCR)

DNA was extracted from samples collected at different time points from the same leaf on MCLV vII-positive plants and used as templates for quantitative real-time PCR (qPCR). The increase in MCLV vII coat protein (CP) copy number was used as an indicator of MCLV vII virus accumulation level. MCLV vII CP copy numbers were determined by SYBR Green I-based absolute qPCR as described by Sun et al.(Sun et al., 2020). Briefly, the full-length CP was amplified by PCR using MCLV CP F/MCLV CP R primers (Tables S1), cloned and inserted into a pMD19-T vector (TaKaRa), and sequenced. The recombinant plasmids containing MCLV vII CP were extracted and quantified using a Nanodrop spectrophotometer (Thermo Fisher Scientific, Waltham, MA, USA). Tenfold serially diluted concentrations of the plasmid stocks with the measured concentrations were used to generate the standard curves through qPCR using a 2×NovoStartR SYBR qPCR Super Mix Plus Kit (Novoprotein, Shanghai, China) on a QuantStudio™3 Flex System (Applied Biosystems, Foster City, CA, USA). The optimized primer pair was qMCLV F/qMCLV R (Tables S1), which targeted the region of the putative CP ORF of MCLV vII. The absolute amounts of MCLV vII CP in the analyzed samples were determined by inserting the Ct values into the standard curve (Figs. 3a). The ratios of the number of MCLV vII CP copies detected in different sample plants inoculated at different stages to the number of MCLV vII CP copies detected in the initial sample plants inoculated with WT were used to represent the relative accumulation of MCLV vII. Three biological replicates were conducted for all qPCR experiments.

## 3 Results

### 3.1 The *V4* and *V5* genes of MCLV vII could be expressed during viral infection

To confirm the presence of MCLV vII V4 and V5 during MCLV infection, specific polyclonal antibodies against V4 and V5 were prepared. However, we failed to detect the presence of V4 and V5 proteins by Western blot after three attempts. To address this conundrum, we detected the presence of V4 and V5 mRNA during virus infection and tested the promoter activity of the upstream sequence of these two ORFs.

**Fig. 2:**
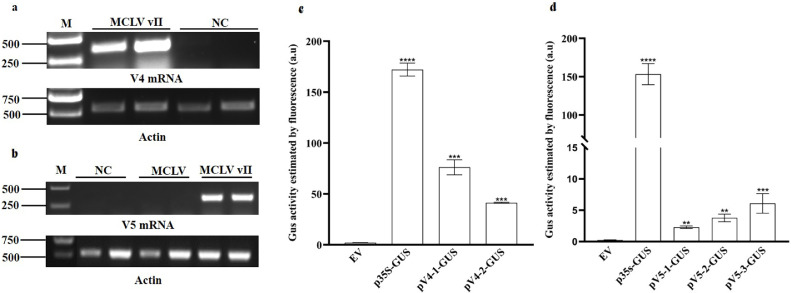
V4 and V5 were expressed during MCLV vII infection. (**a**) RT‒PCR analysis of V4 mRNA in MCLV vII-infected or healthy mulberry plants. Total RNA extracted from MCLV vII-infected and healthy plants was digested by DNase I and then reverse transcribed with *V4* gene-specific primers. (**b)** RT‒PCR analysis of V5 mRNA from MCLV vII-infected or healthy mulberry plants. Total RNA extracted from MCLV-, MCLV vII-infected and healthy plants was DNase I digested and then reverse transcribed with *V5* gene-specific primers. **(c)** and (**d)** Quantitative analysis of V4 and V5 promoter activity. The promoter activity was estimated by the measured fluorescence value of GUS in the different samples. Data are the mean of three independent biological replicates. Bars represent the mean ± standard deviation (SD). Asterisks indicate a statistically significant difference according to unpaired Student's t-test (two-tailed), ** *p* < 0.01, *** *p* < 0.001, **** *p* < 0.0001. M: DNA ladder marker. NC: negative control.

RT‒PCR analysis for the presence of V4 and V5 mRNAs during virus infection was performed using DNase I-digested RNA extracted from MCLV vII-infected mulberry plants as templates and individual specific gene primers. V4 mRNA was detectable in the MCLV-infected but not in the mock-inoculated mulberry plants (Figs. 2a). V5 mRNA could be detected in MCLV vII-infected but not in MCLV- and mock-inoculated mulberry plants (Figs. 2b).

The results of GUS fluorometric assays indicated that the promoter activities of pV4–1 and pV4–2 increased 70 and 40 times compared with that of the empty vector (pCambia1391z), respectively (Figs. 2c). When compared with the negative control, pV5–1, pV5–2, and pV5–3 displayed promoter activities that differed by 2–5 times (Figs. 2d). These results demonstrated that the upstream sequences of V4 and V5 ORFs have promoter activity with different intensities, despite relatively weak activity compared to the positive control 35S. Thus, we speculated that the upstream sequences of the V4 and V5 ORFs could drive the expression of the respective coding regions during viral infection.

Taken together, these results confirmed that the V4 and V5 ORFs of MCLV vII are expressible *in planta*.

### 3.2 MCLV vII-encoded V4 enhances viral replication

**Fig. 3:**
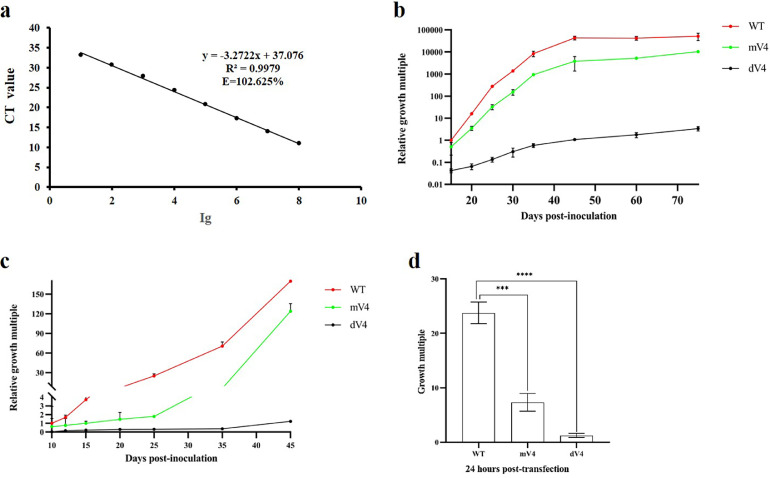
Real-time fluorescence quantitative PCR (qPCR) detection of MCLV replication in plants and protoplast cells. **(a)** Standard curve of qPCR. **(b)** qPCR analysis of MCLV accumulation in systemic leaves of mulberry plants infected with pCA-1.1MCLV^WT^ (WT), pCA-MCLV^mV4^ (mV4), and pCA-MCLV^dV4^ (dV4). **(c)** qPCR analysis of MCLV accumulation in systemic leaves of tomato plants infected with WT, mV4, and dV4. **(d)** qPCR analysis of MCLV replication in protoplast cells transfected with WT, mV4, and dV4. The columns represent the growth multiple calculated by formular of copy number of 24 h −0 h/0 h posttransfection in different samples. Data are the mean of three independent biological replicates. Error bars represent standard deviation (SD). Asterisks indicate a statistically significant difference according to unpaired Student's t-test (two-tailed), *** *p* < 0.001, **** *p* < 0.0001.

To investigate whether MCLV vII-encoded V4 participated in viral infection, *Agrobacterium*-mediated infectious clones (pCA-MCLV^mV4^ and pCA-MCLV^dV4^) of mutant viruses MCLV^mV4^ and MCLV^dV4^ were constructed, and these two infectious clones, as well as the infectious clone (pCA-1.1MCLV^WT^) of wild-type MCLV^WT,^ were agroinoculated into mulberry and tomato plants, respectively. The levels of viral accumulation in plants were assessed by quantifying the copy number of the MCLV vII *CP* gene using qPCR. The accumulation levels of the mutant and wild viruses rose with increasing incubation time in each individual inoculated plant, but the magnitude of the increase varied considerably. The accumulation levels of both mutant viruses in the tomato and mulberry plants inoculated with pCA-MCLV^mV4^ and pCA-MCLV^dV4^ were lower than those of wild-type virus in plants inoculated with pCA-1.1MCLV^WT^. Interestingly, MCLV^dV4^ displayed an obvious reduction in viral multiplication compared to MCLV^mV4^ (Figs. 3). More specifically, MCLV^WT^, MCLV^mV4^ and MCLV^dV4^ were detected in individual agroinoculated mulberry plants at 15 days postinoculation (dpi). There was a 100,000-fold increase in the accumulation level of MCLV^WT^ at 75 dpi compared to 15 dpi, a 10,000-fold increase in the accumulation level of MCLV^mV4^, and only a 5-fold increase in MCLV^dV4^ (Figs. 3b). In agroinoculated tomato plants, MCLV^WT^, MCLV^mV4^ and MCLV^dV4^ were detected at 10 dpi. The accumulation level of MCLV^WT^ at 45 dpi was 150-fold higher than that at 10 dpi, MCLV^mV4^ was 120-fold higher, and MCLV^dV4^ was only threefold higher (Figs. 3c). There was a similar increasing tendency of viruses in agroinoculated tomato plants to that in mulberry plants, although the accumulation rate of MCLV vII was much lower in the experimental host tomato than in the natural host mulberry plants. These results indicated that MCLV vII genome DNA propagation was significantly influenced by the expression of V4 in the host mulberry and experimental tomato plants.

To confirm that the replication of the MCLV^mV4^ and MCLV^dV4^ genomes is reduced compared to that of MCLV^WT^ at the cellular level, we performed protoplast experiments to determine the variations in viral reproduction in cells. The level of viral accumulation in cells was assessed by quantifying the copy number of the MCLV vII *CP* gene using qPCR at 0- and 24-hour posttransfection (hpt) with MCLV^WT^, MCLV^mV4^ and MCLV^dV4^, respectively. There was a *ca.* 24-fold increase in the accumulation level of MCLV^WT^ at 24 hpi compared to 0 hpi, whereas the mutant MCLV^mV4^ has a *ca.* 7-fold increase, MCLV^dV4^ has only a *ca.* 1-fold increase. within protoplast cells (Figs. 3d). The results indicated that MCLV vII genome DNA propagation was significantly influenced by the expression of V4 in protoplast cells.

Symptom observation indicated that neither MCLV^WT^ nor MCLV^mV4^- and MCLV^dV4^-infected mulberry and tomato plants displayed visible typical symptoms (mosaic, crinkle, and rolling), suggesting that the expression of V4 may not be correlated with symptom induction.

The genome sequence analysis of the progeny viruses in the systemic leaves of the inoculated mulberry plants at 75 dpi and tomato plants at 45 dpi determined that MCLV^mV4^ derived from the construct pCA-MCLV^mV4^, MCLV^dV4^ from pCA-MCLV^dV4^, and MCLV^WT^ from pCA-1.1MCLV^WT^.

### 3.3 V5 plays an important role in the infection of MCLV vII into *N. benthamiana*

**Fig. 4:**
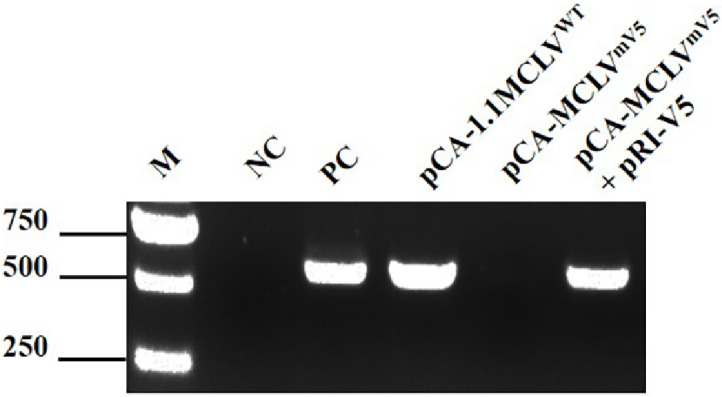
PCR detection for the presence of MCLV vΠ in systemic leaves of *N. benthamiana* at 15 dpi. M: DNA ladder marker. NC: negative control (*N. benthamiana* plants inoculated with empty vector pCAmbia2300). PC: Positive control (mulberry naturally infected with MCLV vΠ).

According to previous studies, there is one MCLV variant that cannot encode the V5 ORF because the nonsense mutation occurring in the V5 ORF could not infect *N. benthamiana* (Lu et al., 2022; Yu 2021). In contrast, MCLV vII, which expresses V5, infects *N. benthamiana*. To investigate whether the *V5* gene is involved in MCLV vII infection of *N. benthamiana*, we agroinoculated pCA-MCLV^mV5^ and pCA-1.1MCLV^WT^ into *N. benthamiana* plants, respectively. Systemic infection of *N. benthamiana* plants (15 dpi) with MCLVWT was determined by PCR, and the results showed that forty-four (73.3%) of 60 *N. benthamiana* plants inoculated with pCA-1.1MCLV^WT^ were MCLV vII-positive, while all 50 *N. benthamiana* plants inoculated with pCA-MCLV ^mV5^ were MCLV-negative. The inoculation experiments were repeated 4 times, and the final statistical results are shown in Table 1 and Figs. 4.

**Table 1:** Viral presence was tested by PCR 15 dpi using DNA extracted from systemic leaves.

	Viral construct inoculated			
	pCA-1.1MCLV^WT^	pCA-MCLV^mV5^	pCA-MCLV^mV5^+ pRI-V5	Negative control
Viral DNA detection of systemic leaves	(44/60) 73.3%	(0/50) 0	(37/60) 61.6%	(0/20) 0

We coinfiltrated pCA-1.1MCLV^mV5^ and pRI-V5 into *N. benthamiana* and repaired the mutant MCLV^mV5^ by a homologous recombination repair mechanism to restore it to wild-type MCLV vII in plants. The progeny viruses and their genomic sequence in the systemic leaves (15 dpi) of *N. benthamiana* plants coinfiltrated with pCA-1.1MCLV ^mV5^ and pRI-V5 were determined by PCR detection and sequencing analysis, respectively. The results showed that thirty-seven (61.6%) of 60 *N. benthamiana* plants coinfiltrated were MCLV vII-infected. The inoculation experiments were conducted 4 times, and the final statistical results are shown in Tables 1 and Figs. 4. To confirm the recovery of ORF5 within the progeny virus genome within systemic leaves of MCLV vII -positive *N. benthamiana* plants, PCR was performed to amplify the ORF5 region using a pair of primer MCLVdORF5 F/MCLVdORF5 R (Tables S1) designed based on the flanking sequences of ORF5. The PCR products were cloned and sequenced. Sequencing results revealed the presence of the V5 ORF (309 nt in size, same as MCLV vII) in the DNA genome of the progeny MCLV vII in systemic leaves of MCLV vII -positive *N. benthamiana* plants. Additionally, the nucleotide sequence of the recovered V5 ORF was identical to that of MCLV vII used to construct pCA-1.1MCLV^WT^. Thus, coinfiltration of pCA-MCLV^mV5^ and pRI-V5 could repair mutant MCLV^mV5^, demonstrating the feasibility of the homologous recombination repair mechanism.

These results indicated that the *V5* gene is essential for MCLV vII to infect *N. benthamiana.*

## 4 Discussion

The genome of MCLV vII presumably encodes seven proteins (Yu 2021). Although previous studies have demonstrated that V4 (Lu et al., 2019; Lu et al., 2020) and V5 (Figures S2) (unpublished, Lu et al.) function as neither posttranscriptional gene silencing suppressors nor pathogenic determinants, their roles during MCLV infection remain poorly understood. In this study, we explored the functions of the *V4* and *V5* genes. According to our results, MCLV^mV4^ and MCLV^dV4^ have no effect on virus infectivity but significantly reduce genome DNA replication at either the plant or cellular level, suggesting that the *V4* gene is crucial to viral DNA replication. It is similar to *begomovirus* C3 protein, which is a replication enhancer protein (REn) and is needed for efficient viral DNA multiplication (Li et al., 2019a; Sunter et al., 1990). MCLV^mV5^ does not destroy infectivity to mulberry but results in disabled infection to *N. benthamiana* plants, which is consistent with a prior report where an MCLV without the V5 ORF could not infect *N. benthamiana* plants (Lu et al., 2022). Therefore, we determined that the *V5* gene is essential for MCLV infectious clones to infect *N. benthamiana* plants. Viral proteins are generally multifunctional and serve a variety of purposes. However, we demonstrate a few functions of V5 and V4 here. Whether they have other functions needs to be clarified. Additionally, more research is still needed to fully understand how V4 influences genome DNA replication during the MCLV life cycle.

Both mutation and deletion of the *V4* gene can cause a decline in replication of the MCLV genome. However, the decrease caused by *V4* gene deletion is much greater than that caused by *V4* gene mutation. We speculated several possible reasons for this result. First, mRNAs of geminiviral genes have been reported to contain a poly (A) tail at the 3′-terminus (Baliji et al., 2007; Frischmuth et al., 1991; Vargas-Asencio et al., 2019). Although it is not yet clear whether the mature mRNAs of MCLV genes contain a poly(A) tail, a search revealed a poly(A) signal sequence (GATAAA) for plant genes downstream of the coding region of MCLV C2, which is closely associated with replication of the MCLV genome. Deletion of the poly(A) signal for C2 in MCLV^dV4^ impaired the formation of mature C2 mRNA and in turn decreased MCLV genome replication. Additionally, we cannot exclude the possibility that the deleted 315-nt sequences may also contain other control elements that could affect viral infectivity/replication. Second, geminivirus-encoded proteins were identified previously using the criterion for an arbitrary threshold of 10 kDa. However, the latest studies found that some small proteins (below 10 kDa) play a biological function during the infection of geminiviruses (Gong et al., 2021). By using NCBI ORFfinder (https://www.ncbi.nlm.nih.gov/orffinder/), we searched for one more ORF (273 nt, 10.2 kDa) embedded in the V4 ORF. The ORF might be expressed as a protein in viral infection and involved in viral replication. Thus, deletion of both ORFs in MCLV^dV4^ was responsible for the significant decrease in MCLV genome replication.

Tomato leaf curl Yunnan virus (TLCYnV) is a recombinant virus derived from tomato yellow leaf curl China virus (TYLCCNV). However, TLCYnV can systematically infect and induce typical symptoms in *N. benthamiana* plants, while TYLCCNV cannot. TLCYnV contains one more C4 protein than TYLCCNV based on sequence alignment. It is proposed that begomovirus C4 could expand the host range and promote the systemic infection of TLCYnV (Cui et al., 2004; Fondong 2019; Hanley-Bowdoin et al., 2013; Hu et al., 2020; Li et al., 2020; Medina-Puche et al., 2021; Zhou et al., 2003). Given the similarities between begomovirus C4 and MCLV vII V5, we hypothesized that V5 could also expand the host range and promote MCLV systemic infection. However, the precise function and mechanism of the V5 protein in the MCLV vII infection process in *N. benthamiana* remain to be determined.

Homologous recombination repair (HRR) is one of the core repair methods for DNA double-strand damage. Site-specific gene correction techniques developed in recent years, such as small fragment homologous replacement (SFHR), use homologous recombination repair mechanisms to repair DNA damage in cells by introducing correct target homologous DNA fragments of defective genes (Yu et al., 2023). One of the main obstacles to HRR in plant species is delivering donor repair templates (DRT) into the nucleus efficiently (Li et al., 2019b). *Agrobacterium*-mediated transient expression can form transferring DNA (T-DNA) containing the target gene, which can enter the nucleus to provide DRT for HRR (Hiei et al., 1994; Li et al., 2019b; Puchta 2010). In this study, MCLV^mV5^ and pRI-V5 were coinfiltrated into *N. benthamiana* to recover the MCLV^mV5^ mutant by using SFHR. The results clearly showed that the progeny viruses in the systemic leaves of the coinoculated *N. benthamiana* recovered to wild-type MCLV vII. Previous studies on the function of viral proteins usually employed transgenic technology to obtain transgenic plants that can express the studied proteins to complete the compensation experiment. We used the homologous recombination repair mechanism to restore the mutational virus to the wild-type virus by coinfecting *N. benthamiana* successfully. Compared with the traditional transgenic compensation experiment, this method is simple and saves time, providing an alternative method for further study of viral protein function.

## Conclusions

In this study, we demonstrated that the *V4* and *V5* genes encoded by MCLV vII could be transcribed and expressed normally during MCLV vII infection in plants. The *V4* gene enhances viral replication, and the V5 gene is needed for MCLV infection of *N. benthamiana*.

## Ethics approval and consent to participate

This article does not contain any studies with human participants or animals performed by any of the authors.

## Consent for publication

Not applicable

## Funding

This work was supported by a grant from the National Natural Science Foundation of China (32271885) and the Doctor Startup Fund Program of Jiangsu University of Science and Technology (No. 1102932301).

## Authors' contributions

QYL, TTH, and JXT conceived and designed the experiments. PZ, PYH, ZNY, YM and JZ cultivated the mulberry, tomato and *N. benthamiana* plants. QYL and TTH wrote the paper. MF revised the paper. All of the authors read and approved the final manuscript.

## CRediT authorship contribution statement

**Tao-Tao Han:** Writing – original draft. **Jia-Xuan Tang:** Writing – review & editing. **Miao Fang:** Writing – review & editing. **Peng Zhang:** Software. **Pei-Yu Han:** Data curation. **Zhen-Ni Yin:** Data curation. **Yu Ma:** Software. **Jian Zhang:** Writing – review & editing. **Quan-You Lu:** Conceptualization, Writing – review & editing.

## Declaration of Competing Interest

The authors declare that they have no known competing financial interests or personal relationships that could have appeared to influence the work reported in this paper.

## Data Availability

Data will be made available on request.

## Appendix Supplementary materials

Image, image 1

Image, image 2

Image, application 3
