# Supplementary figures and images for: Two genes encoded by mulberry crinkle leaf virus (MCLV): The V4 gene enhances viral replication, and the V5 gene is needed for MCLV infection in Nicotiana benthamiana
Source: Virus Res. 2023 Dec 6;339:199288. doi: 10.1016/j.virusres.2023.199288 (PMC10751690; doi:10.1016/j.virusres.2023.199288)

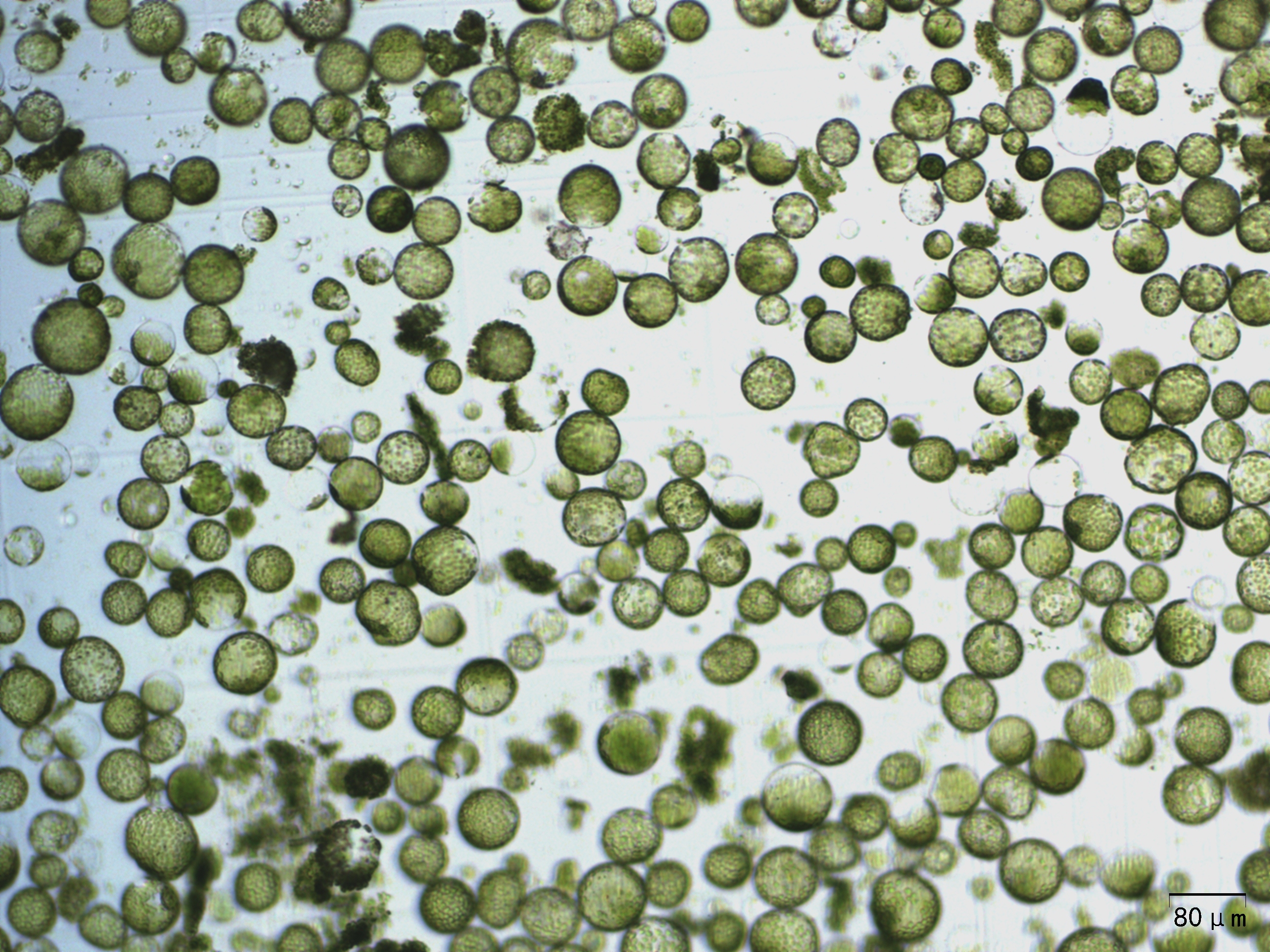

Supplement: Supplementary file 1 [file mmc1.jpg]

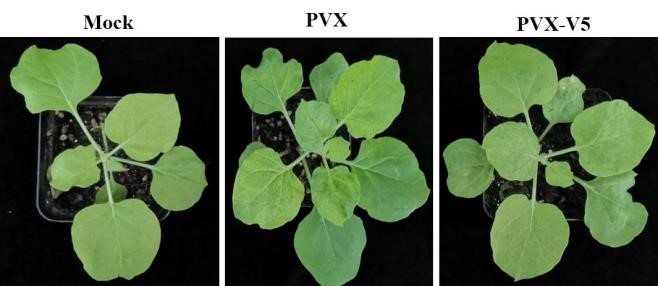

Supplement: Supplementary file 2 [file mmc2.jpg]
