# Supplementary material for: Two genes encoded by mulberry crinkle leaf virus (MCLV): The V4 gene enhances viral replication, and the V5 gene is needed for MCLV infection in Nicotiana benthamiana
Source: Virus Res. 2023 Dec 6;339:199288. doi: 10.1016/j.virusres.2023.199288 (PMC10751690; doi:10.1016/j.virusres.2023.199288)
Supplement: Supplementary file 3 [file mmc3.docx]

Table 1 Primers used in this study and their sequences.

| Primer name | Primer sequence（5′-3′） | Tm/℃ |
| --- | --- | --- |
| pCA V5p F1 | CAAGCTTGGCTGCAGGTCGACATGTTTTCAAGGAGAAAAAAAGAGAA | 53 |
| pCA V5p F2 | CAAGCTTGGCTGCAGGTCGACAATCCGGATCCGTTAGATCTAATT |  |
| pCA V5p F3 | CAAGCTTGGCTGCAGGTCGACCCCTGTTACTGGGTCGGGA |  |
| pCA V5p R | AATTCCCGGGGATCCGTCGACTACCGCCGAAGAAGTATTCCTT |  |
| V5 F | TTGATACATATGCCCGTCGACATGGTCTCTAGCGAAACCGGT | 53 |
| V5 R | TCCGGTACCCCCGGGGTCGACTTAGAAACAAGAAATCCTAATTTCTTT |  |
| MCLV- F1 | CGGGGATCCTCTAGAGTCGACTTGTAGAAGGGAAGGAGTTGGAA | 53 |
| MCLV-mV5 R1 | TCGCTAGAGACCACTACCGCCGAAGAAGTATTCCTT |  |
| MCLV-mV5 F2 | GTAGTGGTCTCTAGCGAAACCGGTAATCGATG |  |
| MCLV-R2 | AGGGCATGCCTGCAGGTCGACCGGTTTCTTGCTCCGCAA |  |
| pCA V4p F1 | CAAGCTTGGCTGCAGGTCGACATGGCGATTACCAGGAGCTCT | 53 |
| pCA V4p F1 | CAAGCTTGGCTGCAGGTCGACATTTGGAATTAAATTAGCCAGTATAAATC |  |
| pCA V4p R | AATTCCCGGGGATCCGTCGACTCAAGTTAATCTGTAAAAAAAATTATAAC |  |
| V4 F | ATGTTTTCAAGGAGAAAAAAAGAG | 57 |
| V4 R | TTAGTTTATTACATGTCTACTCGTG |  |
| MCLV-mV4 R1 | CCTTTTCTCAAGTTAATCTGTAAAAAAAATTATAACTTTT | 53 |
| MCLV-mV4 F2 | CAGATTAACTTGAGAAAAGGTTGTTTTTTCATTAATAGTTCT |  |
| MCLV-dV4 R1 | GGCCTACTAGTACGCGTTAATTAATCAAGTTAATCTGTAAAA | 53 |
| MCLV-dV4 F2 | TTAACGCGTACTAGTAGGCCTGAAAAGGTTGTTTTTTCA |  |
| MCLV jcF | CAGTGAAACGTGGAACATTTGGAAT | 61 |
| MCLV jcR | CAATTAGCAGTCAACGTCACATTCT |  |
| MCLVdORF5 F | TAAGGAATACTTCTTCGGCGGT | 55 |
| MCLVdORF5 R | GTCATTTTCATGCTATTGTCC |  |
| MCLV CP F | ATGGCGATTACCAGGAGCTCTGC | 57 |
| MCLV CP F | TTATTCTGCGTCATAAAAATAAA |  |
| qMCLV F | GCAAGTGTCATGCGAACCCC | 60 |
| qMCLV R | TACCCATTACCACCAGTATG |  |

The sequence with horizontal line is the homologous arm sequence for homologous recombination between target gene and vector. The sequences with wavy line are the homologous arm sequence for homologous recombination between target gene fragments.
